# Supplementary material for: Mechanisms governing the pioneering and redistribution capabilities of the non-classical pioneer PU.1
Source: Nat Commun. 2020 Jan 21;11:402. doi: 10.1038/s41467-019-13960-2 (PMC6972792; doi:10.1038/s41467-019-13960-2)
Supplement: Supplementary file 7 — Source data [file 41467_2019_13960_MOESM7_ESM.zip › Source_Data/FigureS2/FigureS2B_MotifScanOutput/KmeansCluster4/homerResults/motif23.similar.html]

motif23

## Information for motif23

A
G
T
C
A
G
C
T
G
A
T
C
G
T
C
A
A
G
T
C
G
A
C
T
T
C
G
A
G
A
C
T
A
G
T
C
G
T
C
A
A
G
T
C
C
T
A
G
C
T
G
A
  
Reverse Opposite:  

G
A
C
T
G
A
T
C
C
T
A
G
C
A
G
T
T
C
A
G
C
T
G
A
A
G
C
T
C
T
G
A
T
C
A
G
A
C
G
T
C
A
T
G
C
T
G
A
T
C
A
G
  

|  |  |
| --- | --- |
| p-value: | 1e-50 |
| log p-value: | -1.166e+02 |
| Information Content per bp: | 1.752 |
| Number of Target Sequences with motif | 116.0 |
| Percentage of Target Sequences with motif | 2.57% |
| Number of Background Sequences with motif | 131.8 |
| Percentage of Background Sequences with motif | 0.32% |
| Average Position of motif in Targets | 106.8 +/- 49.3bp |
| Average Position of motif in Background | 94.0 +/- 75.8bp |
| Strand Bias (log2 ratio + to - strand density) | 0.4 |
| Multiplicity (# of sites on avg that occur together) | 1.00 |
| Motif File: | file (matrix) reverse opposite |

### Similar de novo motifs found

|  |  |  |  |  |  |  |  |
| --- | --- | --- | --- | --- | --- | --- | --- |
| Rank | Match Score | Redundant Motif | P-value | log P-value | % of Targets | % of Background | Motif file |
| 1 | 0.885 | C G A T T C A G C T G A A G C T C G T A T A C G C G A T A C T G C T G A T C A G G C A T | 1e-44 | -103.551435 | 2.06% | 0.21% | motif file (matrix) |
| 2 | 0.848 | T A G C C G T A A G T C A C G T A G T C C G T A A T G C C G A T T C G A A C G T A G T C C G T A | 1e-42 | -97.171798 | 1.86% | 0.18% | motif file (matrix) |
| 3 | 0.923 | A C G T A T C G C G T A A C G T C G T A A C T G A C G T A T C G C G T A C T A G | 1e-36 | -83.693557 | 2.15% | 0.33% | motif file (matrix) |
